# A Survey on the Side Effects of Pfizer/BioNTech COVID-19 Vaccine Among Vaccinated Adults in Saudi Arabia

**DOI:** 10.7759/cureus.19222

**Published:** 2021-11-03

**Authors:** Rehab A Mohammed, Rana M Garout, Sherehan Wahid, Fatema Ayub, Leen M Firas ZinAlddin, Intessar Sultan

**Affiliations:** 1 Internal Medicine, Ibn Sina National College for Medical Studies, Jeddah, SAU; 2 Internal Medicine, Al-Azhar University Faculty of Medicine for Girls, Cairo, EGY; 3 Medicine, Ibn Sina National College for Medical Studies, Jeddah, SAU

**Keywords:** side effects, herd immunity, covid-19, world pandemic, pfizer-biontech vaccine

## Abstract

Background: Pfizer-BioNTech vaccine was the first of all coronavirus disease (COVID) vaccines to be used in Saudi Arabia. There have been over 17 million doses already administered to the general public in order to successfully reach herd immunity.

Objective: The study aimed to explore the side effects of the Pfizer-BioNTech vaccine.

Materials and methods: This is a cross-sectional study comprising a sample of 386 participating adults of different age groups and genders. A validated modified questionnaire was distributed as a Google form to residents of the kingdom via social networking sites from February to March 2021. The questionnaire included questions regarding participants’ socio-demographic details, vaccination details, and symptom analyses items.

Results: The most common to least reported symptoms were local pain (79.3%), fatigue (42%), muscle pain (39.1%), local swelling (27.7%), joint pain (23.1%), headache (21.8%), fever (21.0%), chills (15.5%), local redness (14.8%), nausea (7.3%), with no reports of anaphylaxis, facial paralysis or syncope. There were more side effects after the second dose than the first (p<0.001). Significant predictors of a higher number of side effects after both doses of the vaccine were the female gender ((p<0.001)) and the presence of allergies (p=0.044).

Conclusion: Pfizer/BioNTech vaccination was quite safe with no reported anaphylaxis or serious events. The most common reported side effects were local pain and fatigue. Symptoms began within 24 hours and were mild to moderate in nature with a regressive course, especially after analgesics. More side effects were experienced after the second dose than the first. The significant predictors of side effects were the female gender and a history of allergies.

## Introduction

Upon declaration of the severe acute respiratory syndrome coronavirus 2 (SARS-CoV-2) as a global pandemic in March 2020 [1], the world has been desperate for a cure as millions suffered from the coronavirus disease 2019 (COVID-19) and its consequences. As of March 12, 2021, the Kingdom of Saudi Arabia reported more than 380,000 cases with a death toll reaching more than 6,000 [2]. With rising cases, nations worldwide reallocated funds to accelerate vaccine production. Successfully, numerous companies managed to produce an efficient and safe vaccine in record time. By March 2021, 12 vaccines were approved for public distribution, which included Pfizer-BioNTech (BNT162b2 mRNA) that has successfully completed phase III of vaccine trials [3]. This was achieved by superimposing the different phases in vaccine trials, reducing the time while maintaining efficacy. Unfortunately, the shorter production time has raised doubts about the safety of the vaccines. 

On December 17, 2020, Saudi Arabia began its first vaccination campaign against COVID-19, starting with the Pfizer-BioNTech vaccine, followed by the administration of Oxford-AstraZeneca in February 2021 [4]. Up until June 2021, the Saudi Ministry of Health has managed to administer more than 17 million doses to its residents, and a plethora more have registered, thus showing interest [5]. Despite the encouraging numbers, many are still skeptical and are determined to be more informed about the vaccine and all its probable effects. A small portion has gone as far as to adopt anti-vaccine attitudes [6]. Scientific analysts expected some to reject vaccination, and that is where our role comes in as doctors and scientists. We must educate the people about the vaccine and make them understand that the benefits outweigh the risks, to reach the appropriate percentage of herd immunity. This enforces the need to highlight the ramifications that were encountered in the population vaccinated, which is what our study aimed to achieve by presenting data from adults vaccinated in Saudi Arabia.

A vital step in mass vaccinations is to assure the public by establishing the efficacy and safety of the available vaccine. Therefore, the aim of this study is to report the side effects of the Pfizer-BioNTech vaccine among vaccinated adults in Saudi Arabia and explore determinants and correlations seen among these side effects.

This study abstract was presented at the fifth Ibn Sina National College - Annual Scientific Conference held on the 2nd and 3rd of April 2021 as a Zoom conference and won best oral presentation.

## Materials and methods

A cross-sectional study was conducted in Saudi Arabia between February 2021 and March 2021. The study tool was a Google form questionnaire that was distributed to the public via social networking sites. The sampling method was a non-probability convenient type, and the minimum recommended sample size was 385, determined by the Raosoft formula. The exclusion criteria included those who received the Oxford-AstraZeneca vaccine and those who were unaware of the type of vaccine they took. The questionnaire used in this study was constructed based on a modified version of the Screening Questionnaire for Adult Immunization [7]. A copy of the questionnaire was provided in both Arabic and English languages.

The questionnaire included the personal information on the respondent’s socio-demographic characteristics, general information related to the COVID-19 vaccine, and symptom analysis items regarding the side effects. This included questions about the type of vaccine received, number of doses, health status in terms of medications taken, illnesses, pregnancy status, or vaccines received up to one month before the vaccine. There were 15 side effects in total that were analyzed in this study. Analysis of the symptoms included the history of onset, course, duration, after which dose it was experienced, severity, treatment, and response to treatment.

This study was approved by Ibn Sina National College Institutional Review Board with reference number (022MP14022021). Consent was taken before the subjects responded to the questionnaire, and confidentiality was ensured as there was no access to the study’s data except for those who were authorized.

Statistical analysis

Data analysis was done using Microsoft Excel 2013 with NumXL version 1.66.44235.2 add-in (Spider Financial Corp., IL) and IBM Statistical Package for the Social Sciences, version 26 (SPSS Inc., Chicago, IL). The number and frequency of each side effect and total side effects were calculated for the 15 symptoms after the first, second, and both doses. Median and interquartile range (IQR) were used to describe the duration of the symptoms which showed abnormal distribution according to the Shapiro-Wilk test.

The rest of the data was normally distributed according to the Shapiro-Wilk test, therefore parametric tests were used to compare the variables. An Independent T-test was done to determine the difference in the number of side effects experienced after the first dose of the vaccine and the number of side effects experienced after the second dose of the vaccine.

Linear regression was carried out to determine the predictors of the total number of side effects experienced after both doses of the Pfizer vaccine using the standardized beta was considered as the odds ratio (OR) with its 95% confidence interval (95% CI). The statistical significance was set at p<0.05.

## Results

There were a total of 536 responses, of which 386 took the Pfizer-BioNTech COVID-19 vaccine. The subjects were mainly of Saudi nationality (95.3%) and were almost equal in gender (Table 1). The subjects were 18 years old and above, and categorized into different age groups: 18-29 (3.4%), 30-49 (30.8%), 50-65 (57.3%), and more than 65 years of age (8.5%). Almost a quarter of the participants were healthcare workers.

**Table 1:** Socio-demographical and clinical data of the Pfizer-BioNTech vaccinated adults in Saudi Arabia

		Frequency	Percent
Age Groups	>65	33	8.5
	50-65	221	57.3
	30-49	119	30.8
	18-29	13	3.4
Sex	Male	177	45.9
	Female	209	54.1
Occupation	Hospital worker	97	25.1
	Non-hospital worker	134	34.7
	Non-worker	70	18.1
	Housewife	82	21.2
	Student	3	0.8
Nationality	Saudi	368	95.3
	Non-Saudi	18	4.7
Comorbidities	No comorbidities	159	41.2
	1 comorbidity	135	35.0
	>1 comorbidity	92	23.8
Medication during vaccination	No medication	173	44.8
	Antihypertensive	98	25.4
	Diabetic medication	91	23.6
	Antihistamine	3	0.8
	Immunosuppressive/Anti-cancer	4	1.0
	Others	155	40.2
Allergies	No allergy	353	91.5
	Drug allergy	11	2.8
	Food allergy	13	3.4
	Inhalant allergy	8	2.1
	Other allergies	5	1.3
Illness during vaccination	Respiratory infection	2	0.5
Other vaccines (1 month prior)	Influenza	13	3.4
	Hepatitis B	2	0.5
Reactions to previous vaccines	Allergic reaction	5	1.3

There were 309 (80.1%) participants who completed two doses of the Pfizer-BioNTech vaccine. From the remaining 77 (19.9%) who took only one dose of the vaccine, the majority were waiting for the second dose and one of the 77 participants reported to have had a previous COVID-19 infection six months prior to the vaccine.

The most common to least reported symptoms include the following: local pain at the site of the injection (306, 79.3%), fatigue (162, 42%), muscle pain (151, 39.1%), swelling at the site of the injection (107, 27.7%), joint pain (89, 23.1%), headache (84, 21.8%), fever (81, 21.0%), chills (60, 15.5%), redness at the site of the injection (57, 14.8%), nausea (28, 7.3%), lower limb weakness (12, 3.1%), lymphadenopathy (11, 2.8%), and loss of sensation of the lower limbs (1, 0.26%) (Table 2). No one reported having experienced a severe reaction such as anaphylaxis, syncope, nor facial paralysis after having either dose of the vaccine. There were significantly more side effects after the second dose (n=807) compared to the first dose (n=713), P<0.001.

**Table 2:** The dose and reported side effects of the Pfizer-BioNTech vaccine in vaccinated adults in Saudi Arabia

	Adults who completed only the first dose of the Pfizer-BioNTech vaccine (n=77)	Adults who completed both doses of the Pfizer-BioNTech vaccine (n=309)			The total frequency of reports (n=386)	Percentage (%)
	Reported after the first dose	Reported after the first dose only	Reported after the second dose only	Reported after both doses		
Pain at site of the injection	58	41	51	156	306	79.3
Fatigue	31	14	71	46	162	42.0
Headache	20	13	34	17	84	21.8
Muscle pain	23	21	72	35	151	39.1
Chills	9	6	36	9	60	15.5
Joint pain	15	9	44	21	89	23.1
Fever	8	11	47	15	81	21.0
Swelling	14	21	28	44	107	27.7
Redness	2	10	20	25	57	14.8
Nausea	8	2	15	3	28	7.3
Lymph node swelling	0	1	10	0	11	2.8
Syncope	0	0	0	0	0	0.0
Facial paralysis	0	0	0	0	0	0.0
Lower limb weakness	2	2	8	0	12	3.1
Loss of lower limb sensation	0	1	0	0	1	0.3
Totals	190	152	436	371	1149	
P=0.000						

The onset of the symptoms was reported mostly at 24 hours (66.3%) followed by starting between 24 to 48 hours (23.2%). The intensity was mainly mild (30%) to moderate (57.9%) with only 12.1% reporting severe symptoms. The course of the reported adverse effects was mainly regressive (51.4%) followed by being intermittent (20.8), then continuous (17.2%), and lastly progressive (10.5%). The median duration for all side effects reported was two days (IQR 11). Some vaccinated adults received paracetamol (57.1%) and to a lesser extent non-steroidal anti-inflammatory drugs (NSAIDs) (10.5%) with a good response of 96.3% (Table 3).

**Table 3:** Description of onset, course, duration, and treatment of the side effects of Pfizer/BioNTech COVID-19 vaccine among adults in Saudi Arabia

Number of reactions		Frequency	Percentage (%)
Total number of side effects: Median (range)		3 (11)	
Onset n=1149	<15 min	72	6.3
	<24 hours	762	66.3
	24-48 hours	268	23.3
	>48 hours	47	4.1
Course n=1149	Regressive	591	51.4
	Intermittent	239	20.8
	Continuous	198	17.2
	Progressive	121	10.5
Duration in days: Median (IQR)		2 (11)	
Severity n=998	Mild	299	30.0
	Moderate	578	57.9
	Severe	121	12.1
Treatment n=1079	No treatment	402	37.3
	Paracetamol	616	57.1
	NSAIDs	113	10.5
Response to treatment n=677	Good response	652	96.3
	No response	25	3.7

A linear regression test was carried out using the data of the 309 participants who had both doses of the vaccine to determine whether the independent variables; age, sex, the presence of comorbidities, the use of medications, and the presence of allergies were predictors of the dependent variable; the total number of side effects experienced after both doses of the Pfizer vaccine. Significant predictors of the total number of side effects are female gender (OR 0.258, 95% CI 0.997-2.464, P<0.001) and a personal history of allergies (OR 0.111, 95% CI 0.038-2.747, P<0.05) (Table 4).

**Table 4:** Predictors of the total number of side effects experienced after both doses of the Pfizer vaccine

Model	Standardized Coefficients	p	95.0% Confidence Interval for B	
	Beta Odd Ratio		Lower Bound	Upper Bound
(Constant)		0.013	0.602	5.169
Age	-0.112	0.058	0-.070	0.001
Sex	0.258	0	0.997	2.464
Comorbidities	0.086	0.202	-0.322	1.518
Medications	0.045	0.494	0-.568	1.174
Allergies	0.111	0.044	0.038	2.747
a. Dependent Variable: Total SE				

## Discussion

The Ministry of Health of Saudi Arabia announced that the nationwide administration of the COVID-19 vaccines will be divided into three stages according to priority groups. Stage one included "citizens and residents over 65 years and professionals who are most vulnerable to infection, people who are obese and have a body mass index (BMI) over 40, those who have immune deficiency, such as those who underwent organ transplantation or taking immunosuppressive drugs; and those who have two or more chronic diseases including asthma, diabetes, chronic kidney disease, chronic heart disease including coronary artery disease, and chronic obstructive pulmonary disease, and those with a history of a previous stroke.” Stage two consists of those who are above 50 years of age, medical practitioners, and those who have one of the chronic diseases mentioned above, as well as a BMI of 30-40. Stage three is the remaining individuals who do not fall in the first two stages. In our study, most participants fell under the age group of 50 to 65 years who were targeted in the second stage [8]. Moreover, more than half of the participants had comorbidities and almost a quarter were healthcare workers. Therefore, we could consider our participants as a risky group and interpret results accordingly. 

Localized pain at the site of the injection was observed in over three-quarters of the population making it the most reported side effect, as supported by the Food and Drug Administration (FDA) guidelines set post-vaccine trials [9]. Local pain remains the commonest adverse effect noticed in numerous other injectable vaccine trials carried out previously [10]. In spite of being the most common side effect of intramuscular (IM) vaccines, localized pain was not observed by 80 individuals in our study which could be explained by optimal vaccine administration practices done to reduce reactogenicity [11]. According to an article, headache, arm pain, and fever were self-limited yet some subjects used simple analgesics and antipyretics for relief [12]. The symptoms can therefore be considered mild. To ensure optimal efficacy and minimal adversities, the Centers for Disease Control (CDC) has issued principles for safe vaccine administration [13].

A very small percentage of our sample reported lower limb weakness and there was a single report of lower limb loss of sensation. Previously, there have been few reports of lower limb affection after the first dose of COVID-19 vaccines, consistent with the diagnosis of Guillain Barre syndrome [14-16]. The reports of lower limb affection in our study, however, cannot be associated with this diagnosis since lower limb weakness and loss of sensation were not properly defined to the participants and may have been easily confused with generalized fatigue. When observing the data closely, participants who reported lower limb weakness have also either chosen one or a combination of the following symptoms: generalized fatigue, muscle pain, and/or joint pain. An even smaller percentage of the population noticed lymphadenopathy. Although it has been reported previously in other vaccines such as influenza or human papillomavirus vaccines, it is not as commonly reported as other side effects [17].

There were no reports of syncope or facial paralysis in this study. According to the CDC, syncope is a common occurrence with all vaccines and is not caused by the constituents of the vaccine itself rather by the anxiety and pain while receiving the vaccine. It is also most commonly observed in those aged 11 to 18 years, an age group that was excluded in our study [18]. The absence of reports of facial paralysis is also supportive of another study that stated that even if a correlation existed between the COVID-19 vaccines and facial paralysis, the risk is most likely very low as observed with other viral vaccines [19].

The onset of side effects was most commonly noticed within 24 hours, regressive in nature, and typically mild to moderate in severity. These findings are similar to clinical trials carried out by the CDC and FDA [20]. However, there have been reports of side effects in our study that were continuous in nature, mainly in participants who experienced local pain, muscle pain, and fatigue.

Though there were no reports of anaphylaxis in our study, in order to ensure the health and wellbeing of the vaccine recipients, the ministry of health of Saudi Arabia had protocols published, namely the Saudi MOH Emergency Medical Protocol for Management of Anaphylactic Reaction. Furthermore, many platforms for adverse effects reporting were integrated to appropriately manage any recipients who experienced hypersensitivity reactions before their second dose of the vaccine [21].

It was found that the second dose of the Pfizer-BioNTech vaccine showed a significantly higher number of adverse effects than the first. Information collected by the CDC from Pfizer vaccine recipients in the United States showed that more side effects were reported after the second dose. Similar results were produced by the FDA and other studies [20,22]. However, this should not discourage the public from obtaining the second dose of the Pfizer vaccine and further public reassurance needs to be given that the known benefits of the COVID-19 vaccination outweigh the known risks [23]. Studies suggest that a vaccine-induced antibody response can be observed after the second dose of the Pfizer vaccine for a previously non-infected recipient and after the first dose in a previously infected recipient along with signs of reactogenicity [22].

According to the FDA, younger individuals were more likely to experience more pronounced side effects to the vaccine [20]. After either one of the Pfizer-BioNTech vaccine doses, our study found age as a non-significant negative predictor of the total number of side effects. This may be attributed to a more robust immune system causing a stronger immune response in younger vaccine recipients [24]. By the end of June 2021, the MOH had announced the initiation of Pfizer-BioNTech inoculation of the youngest age group (12-18 years) [25]. This initiative aims to return to in-person schooling by the start of the next academic year [26].

Historically, clinical trials were conducted with men being the dominant gender thereby their reactions were better anticipated by researchers [27]. However, our study consisted of an almost equal number of male and female participants unsupportive of the aforementioned theory. A 2004 study comparing pain perception in men and women concluded that a greater number of women experienced localized pain after injection [28]. The likelihood of local site reactions like pain, redness, and swelling in women is thought to be due to them unable to fully relax before the injection, consequently, the deltoid remains flexed. The female gender was a significant predictor of a higher total number of side effects after both doses of the Pfizer vaccine in this current study. This observation has been well established in multiple studies done on the response to influenza vaccines and is suggested to be associated with elevated antibody response and expression of inflammatory cytokines in females based on androgen levels and genes involved in lipid synthesis [28]. Studies done on influenza vaccine recipients have also proved that females have higher cross-protection against novel influenza viruses when compared to males. Whether female recipients of the Pfizer vaccine show better cross-protection against newer variants of the virus calls for further investigation. Regardless, it is the popular belief that WHO-approved vaccines should provide effective protection against most strains of the SARS-CoV-2 virus due to their ability to provoke a considerably broad immune response [29].

The presence of allergies towards a variety of antigens was also a predictor of a higher number of side effects after the vaccine. Multiple sources recommend that people with a history of severe allergies not specific to any components of the vaccine are still eligible for a vaccination against COVID-19 [30].

## Conclusions

The Pfizer/BioNTech COVID-19 vaccine was quite safe with no reported anaphylaxis or serious events. The most common reported side effects were local pain, fatigue, muscle pain, local swelling, joint pain, headache, fever, chills, local redness, and nausea. Most adverse effects began within 24 hours of having the vaccine and were mild to moderate with regressive nature and good response to analgesics. More side effects were experienced after the second dose than after the first dose. The significant predictors of a higher number of side effects after both doses were the female gender and a history of allergies. Further studies are needed to estimate the potential long-term adverse effects of this vaccine. Other studies are also needed to compare the adverse effects of the Pfizer/BioNTech COVID-19 vaccine with other COVID-19 vaccines.

## References

[1] (2021). WHO timeline - COVID-19. https://www.who.int/news/item/27-04-2020-who-timeline---covid-19.

[2] (2021). Worldometer. https://www.worldometers.info/coronavirus/country/saudi-arabia.

[3] Sonani B, Aslam F, Goyal A, Patel J, Bansal P (2021). COVID-19 vaccination in immunocompromised patients. Clin Rheumatol.

[4] Saudi Arabia vaccinates 300, 000 against COVID-19, no side effects detected [Internet]. Arab News. https://arab.news/cvwyn (2021). Saudi Arabia vaccinates 300,000 against COVID-19, no side effects detected. https://www.arabnews.com/node/1794306/saudi-arabia.

[5] (2021). Saudi Arabia records 1,312 new COVID-19 cases as over 17mn vaccine doses administered in Kingdom. http://saudigazette.com.sa/article/608152..

[6] Fisher KA, Bloomstone SJ, Walder J, Crawford S, Fouayzi H, Mazor KM (2020). Attitudes toward a potential SARS-CoV-2 vaccine: a survey of U.S. adults. Ann Intern Med.

[7] (2021). Immunization Action Coalition (IAC): vaccine information for health care professionals. https://www.immunize.org.

[8] (2021). Ministry Of Health Saudi Arabia. https://www.moh.gov.sa/en/Pages/Default.aspx.

[9] (2021). Comirnaty and Pfizer-BioNTech COVID-19 vaccine. https://www.fda.gov/emergency-preparedness-and-response/coronavirus-disease-2019-covid-19/comirnaty-and-pfizer-biontech-covid-19-vaccine.

[10] Nichol KL, Margolis KL, Lind A (1996). Side effects associated with influenza vaccination in healthy working adults: a randomized, placebo-controlled trial. Arch Intern Med.

[11] Petousis-Harris H (2008). Vaccine injection technique and reactogenicity--evidence for practice. Vaccine.

[12] Polack FP, Thomas SJ, Kitchin N (2020). Safety and efficacy of the BNT162b2 mRNA Covid-19 vaccine. N Engl J Med.

[13] (2021). Interim clinical considerations for use of COVID-19 vaccines currently approved or authorized in the United States. https://www.cdc.gov/vaccines/covid-19/info-by-product/clinical-considerations.html.

[14] Waheed S, Bayas A, Hindi F, Rizvi Z, Espinosa PS (2021). Neurological complications of COVID-19: Guillain-Barre syndrome following Pfizer COVID-19 vaccine. Cureus.

[15] Patel SU, Khurram R, Lakhani A, Quirk B (2021). Guillain-Barre syndrome following the first dose of the chimpanzee adenovirus-vectored COVID-19 vaccine, ChAdOx1. BMJ Case Rep.

[16] (2021). Guillain-Barre after COVID-19 vaccine: case report. https://www.medpagetoday.com/infectiousdisease/covid19vaccine/91969.

[17] Studdiford J, Lamb K, Horvath K, Altshuler M, Stonehouse A (2008). Development of unilateral cervical and supraclavicular lymphadenopathy after human papilloma virus vaccination. Pharmacotherapy.

[18] (2021). Fainting (syncope) after vaccination. https://www.medpagetoday.com/infectiousdisease/covid19vaccine/92369.

[19] (2021). COVID-19 vaccines: facial paralysis / CDC J&J guidance. https://www.jwatch.org/fw117742/2021/04/27/covid-19-vaccines-facial-paralysis-cdc-j-j-guidance..

[20] Wollersheim S (2021). FDA review of efficacy and safety of Pfizer-BioNTech COVID-19 vaccine emergency use authorization request. https://www.fda.gov/media/144337/download.

[21] Assiri A, Al-Tawfiq JA, Alkhalifa M (2021). Launching COVID-19 vaccination in Saudi Arabia: lessons learned, and the way forward. Travel Med Infect Dis.

[22] Ebinger JE, Fert-Bober J, Printsev I (2021). Antibody responses to the BNT162b2 mRNA vaccine in individuals previously infected with SARS-CoV-2. Nat Med.

[23] (2021). Pfizer-BioNTech COVID-19 vaccine overview and safety (also known as COMIRNATY). 20212021.

[24] Giefing-Kröll C, Berger P, Lepperdinger G, Grubeck-Loebenstein B (2015). How sex and age affect immune responses, susceptibility to infections, and response to vaccination. Aging Cell.

[25] (2021). Saudi Arabia to inoculate those aged 12 to 18 with Pfizer vaccine: health ministry. 2021.

[26] (2021). The Minister of Education discusses the final preparations for the start of the school year tomorrow with the education directors. https://www.moe.gov.sa/en/mediacenter/MOEnews/Pages/r-m-2021-76.aspx.

[27] (2021). Lack of females in drug dose trials leads to overmedicated women: gender gap leaves women experiencing adverse drug reactions nearly twice as often as men, study shows. https://www.sciencedaily.com/releases/2020/08/200812161318.htm.

[28] Mencke T, Schreiber JU, Knoll H, Stracke C, Kleinschmidt S, Rensing H, Silomon M (2004). Women report more pain on injection of a precurarization dose of rocuronium: a randomized, prospective, placebo-controlled trial. Acta Anaesthesiol Scand.

[29] (2021). Episode #20 - COVID-19 - variants & vaccines. 2021.

[30] (2021). Vaccines and immunization: what is vaccination?. https://www.who.int/news-room/q-a-detail/vaccines-and-immunization-what-is-vaccination.

